# Mitochondrial defects and metabolic vulnerabilities in Lynch syndrome–associated MSH2-deficient endometrial cancer

**DOI:** 10.1172/jci.insight.185946

**Published:** 2025-02-18

**Authors:** Mikayla Borthwick Bowen, Brenda Melendez, Qian Zhang, Diana Moreno, Leah Peralta, Wai Kin Chan, Collene Jeter, Lin Tan, M. Anna Zal, Philip L. Lorenzi, Kenneth Dunner, Richard K. Yang, Russell R. Broaddus, Joseph Celestino, Nisha Gokul, Elizabeth Whitley, Deena M. Scoville, Tae Hoon Kim, Jae-Wook Jeong, Rosemarie Schmandt, Karen Lu, Hyun-Eui Kim, Melinda S. Yates

**Affiliations:** 1 Department of Gynecologic Oncology,; 2 Department of Genomic Medicine,; 3 Metabolomics Core Facility, Department of Bioinformatics and Computational Biology,; 4 Department of Epigenetics and Molecular Carcinogenesis,; 5 Department of Cancer Biology, and; 6 Department of Pathology, The University of Texas MD Anderson Cancer Center, Houston, Texas, USA.; 7 Department of Pathology & Laboratory Medicine, University of North Carolina at Chapel Hill School of Medicine, Chapel Hill, North Carolina, USA.; 8 Department of Veterinary Medicine, The University of Texas MD Anderson Cancer Center, Houston, Texas, USA.; 9 Department of Obstetrics, Gynecology, and Women’s Health, University of Missouri, Columbia, Missouri, USA.; 10 Department of Integrative Biology and Pharmacology, The University of Texas Health Science Center at Houston McGovern Medical School, Houston, Texas, USA.j

**Keywords:** Cell biology, Oncology, Cancer, Mitochondria, Obstetrics/gynecology

## Abstract

Lynch syndrome (LS), caused by inherited mutations in DNA mismatch repair genes, including *MSH2*, carries a 60% lifetime risk of developing endometrial cancer (EC). Beyond hypermutability, mechanisms driving LS-associated EC (LS-EC) remain unclear. We investigated MSH2 loss in EC pathogenesis using a mouse model (*PR-Cre Msh2^LoxP/LoxP^*, abbreviated Msh2KO), primary cell lines, human tissues, and human EC cells with isogenic MSH2 knockdown. By 8 months, 58% of Msh2KO mice developed endometrial atypical hyperplasia (AH), a precancerous lesion. At 12–16 months, 50% of Msh2KO mice exhibited either AH or ECs with histologic similarities to human LS-ECs. Transcriptomic profiling of EC from Msh2KO mice revealed mitochondrial dysfunction–related pathway changes. Subsequent studies in vitro and in vivo revealed mitochondrial dysfunction based on 2 mechanisms: mitochondrial content reduction and structural disruptions in retained mitochondria. Human LS-ECs also exhibited mitochondrial content reduction compared with non-LS-ECs. Functional studies demonstrated metabolic reprogramming of MSH2-deficient EC, including reduced oxidative phosphorylation and increased susceptibility to glycolysis suppression. These findings identified mitochondrial dysfunction and metabolic disruption as consequences of MSH2 deficiency in EC. Mitochondrial and metabolic aberrations should be evaluated as biomarkers for endometrial carcinogenesis or risk stratification and represent potential targets for cancer interception in women with LS.

## Abstract 2

This study reports mitochondrial dysfunction contributing to MSH2-deficient endometrial cancer development, identifying a noncanonical pathway for MSH2 deficient carcinogenesis which imparts vulnerability to metabolic targeting.

## Introduction

Lynch syndrome (LS) is the most common cancer susceptibility syndrome, affecting 1 in 279 individuals in the United States (1). LS increases the risk of multiple cancer types, most commonly endometrial cancer (EC) and colorectal cancer. LS is an autosomal dominant inherited syndrome defined by a loss-of-function mutation in a DNA mismatch repair (MMR) gene, namely *MLH1*, *MSH2*, *MSH6*, or *PMS2* (2). Loss of DNA MMR, termed mismatch repair deficiency (MMRd), leads to increased DNA mutations and thus increased likelihood for oncogenic mutations to occur. LS is associated with an up to 60% lifetime risk of EC, and approximately 2%–5% of all diagnosed ECs are attributed to LS (3, 4). Major knowledge gaps remain in understanding EC pathogenesis and opportunities for improved risk stratification and cancer interception are critically needed.

Prior studies in colon cancer cells or embryonic stem cells have shown increased susceptibility of MMRd cells to accumulate DNA damage in response to oxidative stress (5–7) and ionizing radiation (8–10), suggesting the propensity for noncanonical consequences of MMRd to influence carcinogenesis. Mouse models with global loss of MMR gene *Msh2*, *Pms2*, or *Mlh1* produce lymphomas at an early age (11–13), which has been a limiting factor to studying tumorigenesis in other tissues. Translational studies on LS-related EC (LS-EC) development have been limited, in part due to lack of a mouse model for LS-EC. We report here a valuable mouse model for LS-EC development through MSH2 loss targeted to the female reproductive tract, which facilitates mechanistic studies and will revolutionize translational research in LS-EC. Elucidating mechanisms of LS-EC development will be critical in providing novel biomarkers for risk stratification and/or therapeutic targets for prevention and interception.

Mitochondrial dysfunction has long been implicated in cancer development and progression due to its effects on metabolic reprogramming, generation of reactive oxygen species, hypoxia signaling, and apoptosis signaling (reviewed in ref. 14). In EC, studies have evaluated the impact of hyperestrogenism on mitochondria. Because estrogen stimulates mitochondrial biogenesis, EC is typically associated with increased mitochondrial content (15). Indeed, type I ECs, an outdated classification of ECs associated with hyperestrogenism, exhibit elevated mitochondrial biogenesis compared with benign endometrium (16). However, a subset of ECs from The Cancer Genome Atlas (TCGA) exhibits transcriptional signatures indicative of low mitochondrial content and function (17). Importantly, these “OXPHOS-low” ECs exhibited significantly increased prevalence of MMRd compared with ECs with intact oxidative phosphorylation signaling. The prior associations of mitochondrial dysfunction with MMRd colorectal cancer (18) and impaired oxidative phosphorylation signaling in MMRd ECs suggest a potential link between LS and/or MMRd and mitochondrial disruption. Our studies probe this critical link in MSH2-deficient EC using a mouse model.

This study reports a valuable mouse model of LS-EC and defines a role for MSH2 loss in mitochondrial defects and metabolic reprogramming. We utilize this mouse model, including multiple cell lines derived from this system, in parallel with cell lines and tissue from patients with LS to interrogate the importance of this phenotype in development of LS-EC. Here, we identify mitochondrial dysfunction as a mechanism for LS-EC development. Mitochondrial dysfunction and metabolic disruption are consequences of MSH2 deficiency–related EC and have important implications for cancer interception or therapeutic approaches for LS-EC. In addition, mitochondrial content loss and dysfunction should be evaluated as potential shared mechanisms for EC development through other risk conditions for EC development.

## Results

### Mouse model for MSH2 loss targeted to the reproductive tract develops endometrial pathology similar to human LS-ECs.

**Figure 1:**
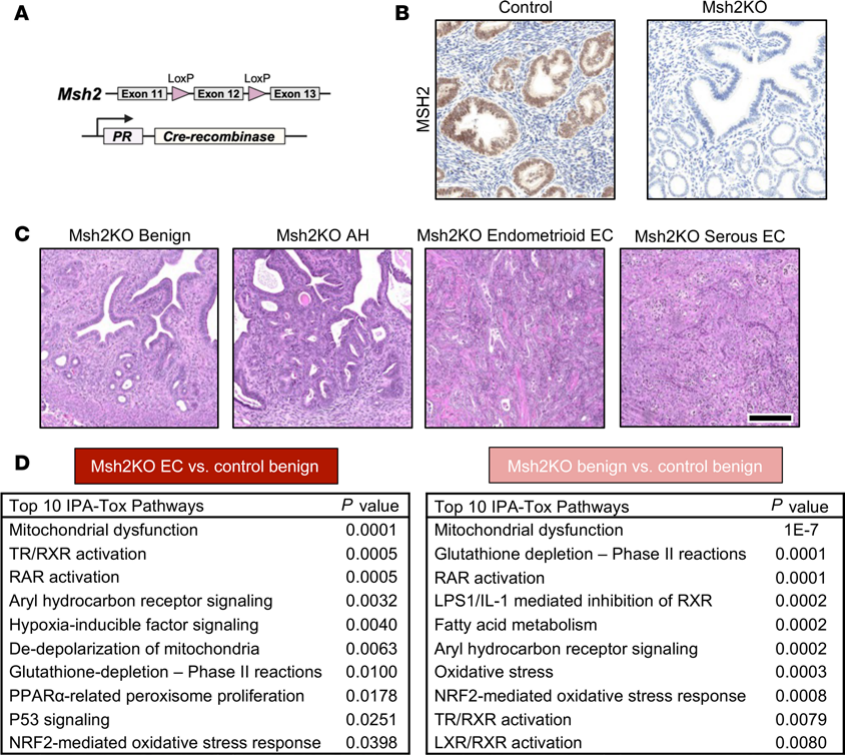
Characterization of mouse model for MSH2-deficient EC development. (**A**) Msh2KO mice express *PR-Cre* and a floxed *Msh2* gene segment. (**B**) Presence of MSH2 by immunohistochemistry (IHC) in control mouse endometrium (left) and Msh2KO mouse endometrium (right). Scale bar: 200 μm. (**C**) Representative images of H&E-stained uterine tissues from Msh2KO mice with benign endometrium, Msh2KO mice with AH, and Msh2KO mice with uterine tumors with endometrioid-like and serous-like histology. Scale bar: 200 μm. (**D**) Top 10 dysregulated pathways in Msh2KO EC (left, *n* = 7) or Msh2KO benign endometrium (right, *n* = 6) versus control benign endometrium (*n* = 4) identified by Ingenuity Pathway Analysis (IPA), which uses right-tailed Fisher’s exact tests using differentially expressed genes identified using the Transcriptome Analysis Console (ANOVA with eBayes analysis correction) from ClariomD microarray transcriptome analyses.

To achieve *Msh2* loss in the endometrium, we bred progesterone receptor (PR) Cre knockin (*PR-Cre*) mice (19) with *Msh2^LoxP/LoxP^* mice (20) to produce *PR-Cre*^+^*Msh2^LoxP/LoxP^* mice with targeted loss of MSH2 expression in tissues that express the PR, including the uterus, as well as very limited expression based on estrus cycle or lactation status in the ovaries and mammary glands (Figure 1, A and B). Female mice expressing *PR-Cre* and *Msh2^LoxP/LoxP^* (hereafter abbreviated Msh2KO) developed endometrial pathology as early as 8 months of age, with 58% of Msh2KO mice exhibiting a precancerous atypical hyperplasia (AH) lesion. By 12 to 16 months of age, 23% of Msh2KO mice developed EC and an additional 27% exhibited AH (Table 1). Msh2KO mice did not develop gross abnormalities in mammary or ovarian tissues. Control mice (*Msh2^LoxP/LoxP^* without *PR-Cre*) did not develop tumors and rarely (7%) developed AH. Msh2KO tumors ranged from microscopic lesions to large tumors involving an entire uterine horn. Tumors that developed in Msh2KO mice exhibited histology similar to human endometrioid (42.9%), serous (42.9%), or mixed endometrioid with serous and/or mucinous (14.3%) subtypes and all were microsatellite unstable. Mutational profiling by whole exome sequencing of 7 tumors that developed in Msh2KO mice is shown in Supplemental Table 1; supplemental material available online with this article; https://doi.org/10.1172/jci.insight.185946DS1 Representative images of mouse tissues throughout the Msh2KO EC development spectrum are shown in Figure 1C.

### MSH2 loss revealed transcriptional signature for mitochondrial dysfunction in Msh2KO mice.

We profiled an initial cohort of Msh2KO tumors (*n* = 7) and Msh2KO mice with benign endometrium (*n* = 6) compared with age-matched control mice (*n* = 4) using transcriptomic profiling of microdissected glands via microarray analysis (Clariom D). A total of 621 genes were differentially expressed between control and Msh2KO benign endometrium, with fold-change values of greater than 2 or less than –2 and a false discovery rate (FDR) of less than 0.05 (Supplemental Table 2). Between control benign endometrium and Msh2KO EC, 2,083 genes were differentially expressed under the same criteria (Supplemental Table 3). Pathway analysis (Ingenuity) of differentially expressed genes revealed 88 dysregulated pathways in Msh2KO benign endometrium and 101 dysregulated pathways in Msh2KO EC compared with control benign endometrium (*P* < 0.05) (Figure 1D). The top pathway significantly differing in both Msh2KO benign endometrium and EC compared with control benign endometrium was “Mitochondrial Dysfunction.” Differentially expressed genes in Msh2KO benign endometrium versus control benign endometrium that met these cutoffs included 31% of genes in the Mitochondrial Dysfunction pathway (48 of 155 total genes in pathway, *P* < 0.0001). Differentially expressed genes in Msh2KO EC versus control benign endometrium that met these cutoffs included 23% of the genes in the Mitochondrial Dysfunction pathway (36 of 155 total genes in pathway, *P* < 0.0001). Altered genes within the Mitochondrial Dysfunction pathway common to both Msh2KO benign and EC in comparison with control benign endometrium included those coding for subunits of all 5 mitochondria electron transport chain complexes and other mitochondrial metabolism–related genes: *Atp5mc3*, *Casp8*, *Cox11*, *Cpt1a*, *Gpd2*, *Mapk9*, *Mtnd2*, *Ndufa8*, *Ndufa9*, *Nudfa13*, *Ndufb9*, *Ndufs6*, *Ogdh*, *Sdhb*, *Ucp2*, *Uqcr11*, *Uqcrc1*, and *Uqcrh* (fold changes of less than –2 and FDR of less than 0.05 for all).

### Msh2KO EC exhibits loss of mitochondrial content.

**Figure 2:**
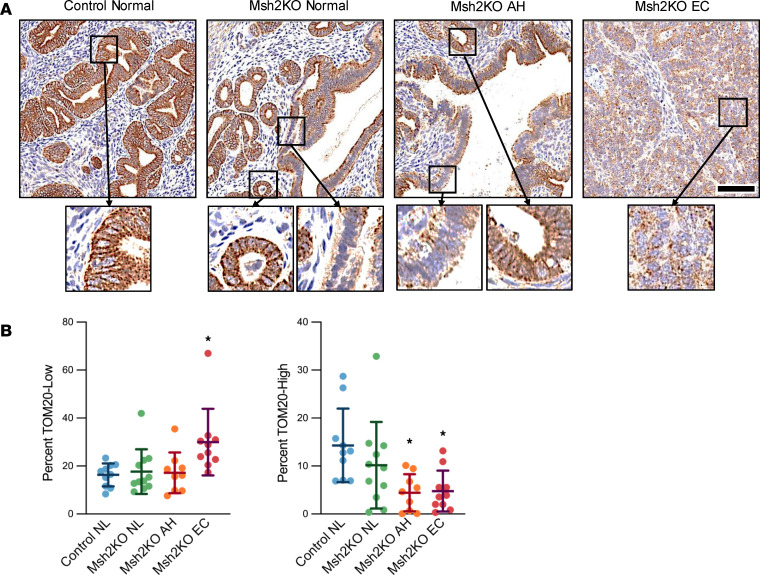
Mitochondrial content decreased during Msh2KO EC development. (**A**) Representative images (original magnification, ×20) of TOM20-stained control normal endometrium (*n* = 10 total analyzed), Msh2KO normal endometrium (*n* = 11 total analyzed), Msh2KO AH (*n* = 9 total analyzed), and Msh2KO EC (*n* = 10 total analyzed) IHC specimens. Scale bar: 100 μm. Insets, ×40 magnification. Staining of glandular areas was quantified using ImageScope Cytoplasmic algorithm. (**B**) Percentage cells with low (left) and high (right) TOM20 staining. Data are mean ± SD. Data points are values from individual mice. Analysis was by ANOVA with Dunnett’s multiple-comparison test. *P < 0.01 compared with control normal (NL) endometrium.

With transcriptomic signatures pointing to mitochondrial dysfunction, including reduced expression of multiple mitochondrial electron transport chain complex genes in MSH2-deficient EC development (see Supplemental Tables 2 and 3), we evaluated overall mitochondrial content at the protein level. Immunohistochemistry (IHC) for the mitochondrial surface marker translocase of the outer mitochondrial membrane complex subunit 20 (TOM20) throughout the EC development spectrum showed that Msh2KO endometrial tumors exhibited significantly reduced TOM20 staining (Figure 2). The percentage of cells with low TOM20 expression (defined as cytoplasm score less than 3+ in ImageScope’s Cytoplasmic v2 algorithm, see Methods for details), indicating low mitochondrial content, was increased in Msh2KO tumor tissue (30%) compared with control (16.3%) (*P* < 0.01). The percentage of cells with high TOM20 expression (defined as cytoplasm score greater than 3+ in ImageScope’s Cytoplasmic v2 algorithm, see Methods for details), indicating high mitochondrial content, was decreased in Msh2KO tumor tissue (4.78%) compared with control endometrium (14.29%) (*P* < 0.01) (Figure 2B). Additionally, the percentage of TOM20^hi^ cells was reduced in the Msh2KO AH tissue (4.43%, *P* < 0.01), indicating that mitochondrial content reduction may begin at the precancerous AH stage. Zoomed insets (×40) show staining pattern differences; whereas control benign endometrium has strong staining throughout the cytoplasm, Msh2KO benign endometrium and AH tissues show patchy areas of low staining. Msh2KO EC shows low staining with focal perinuclear positivity.

**Figure 3:**
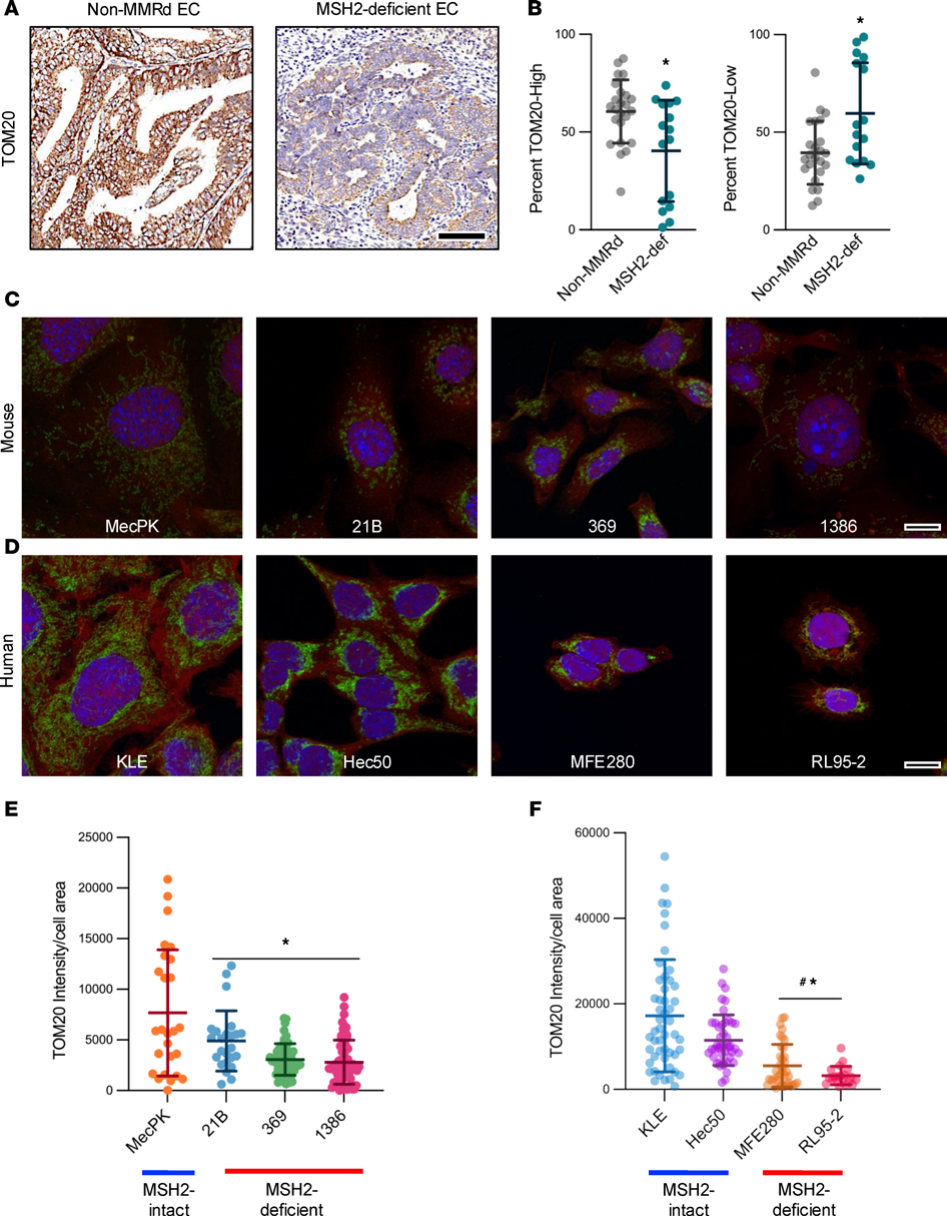
MSH2-deficient EC exhibited mitochondrial content reduction. (**A**) Representative images of TOM20-stained human non-MMRd EC (left, *n* = 22 total analyzed) and MSH2-deficient EC (right, *n* = 13 total analyzed) by IHC. Scale bar: 100 μm. Staining was quantified using ImageScope Cytoplasmic algorithm. (**B**) Percentage cells with high (left) and low (right) TOM20 staining. Data are mean ± SD. Data points are values from individual patient samples. Analysis was by Mann-Whitney *U* test. *P < 0.05 compared with non-MMRd. (**C**) Mouse and human (**D**) EC cell lines were stained using immunofluorescence for TOM20 (green) and HCS CellMask (red) and counterstained with Hoechst 33342. Representative *Z*-stacked Airyscan-processed maximum intensity projection images shown. Scale bars: 10 μm. Mitochondrial content (TOM20 intensity) corrected for cell area (HCS CellMask) was quantified using CellProfiler for mouse (**E**) and human (**F**) EC cell lines. For immunofluorescence studies, each cell line had *n* = 3 technical replicates. Data are mean ± SEM. Data points are values from individual cells. Analysis was by ANOVA with Dunnett’s multiple-comparison test. (**E**) *P < 0.01 relative to MecPK. (**F**) ^#^*P* < 0.0001 relative to KLE; *P < 0.05 relative to Hec50.

To evaluate the relevance to human LS-EC and determine whether mitochondrial content loss is specific to MSH2-deficient EC rather than reflective of carcinogenesis more broadly, we conducted IHC staining against TOM20 in non-MMRd human ECs and those with MSH2 deficiency (Figure 3A). The percentage of TOM20^hi^ cells was reduced by 20.2% and that of TOM20^lo^ cells was increased by 20.2% in MSH2-deficient ECs compared with non-MMRd ECs (Figure 3B), further supporting the notion that mitochondrial content reduction is an MSH2 deficiency–related consequence in EC.

A panel of MSH2-deficient mouse and human cell lines was evaluated using immunofluorescence imaging of TOM20-stained cells. These in vitro studies confirmed that MSH2-deficient mouse and human cells similarly exhibited reductions in mitochondrial content (TOM20 intensity relative to cell size [HCS CellMask]) compared with MSH2-intact counterparts (Figure 3, C–F). Compared with MSH2-intact mouse EC cells (MecPK), the unique EC cell lines derived from 3 different Msh2KO tumors (21B, 369, and 1386) exhibited 36%, 60%, and 64% decreases in TOM20 intensity, respectively (*P* < 0.01). Compared with MSH2-intact human EC cells (KLE), MSH2-deficient MFE280 and RL95-2 cells had 68% and 81% decreases in TOM20 intensity (*P* < 0.0001), signifying that MSH2-deficient mouse and human EC cell lines exhibit mitochondrial content reduction. An additional MSH2-intact human EC cell line (Hec50) had reduced TOM20 intensity compared with KLE cells (by 33%) but had greater TOM20 intensity than both MFE280 and RL95-2 (by 52% and 72%, respectively) (*P* < 0.05).

### MSH2-deficient EC cells exhibit defects in mitochondrial integrity and function.

**Figure 4:**
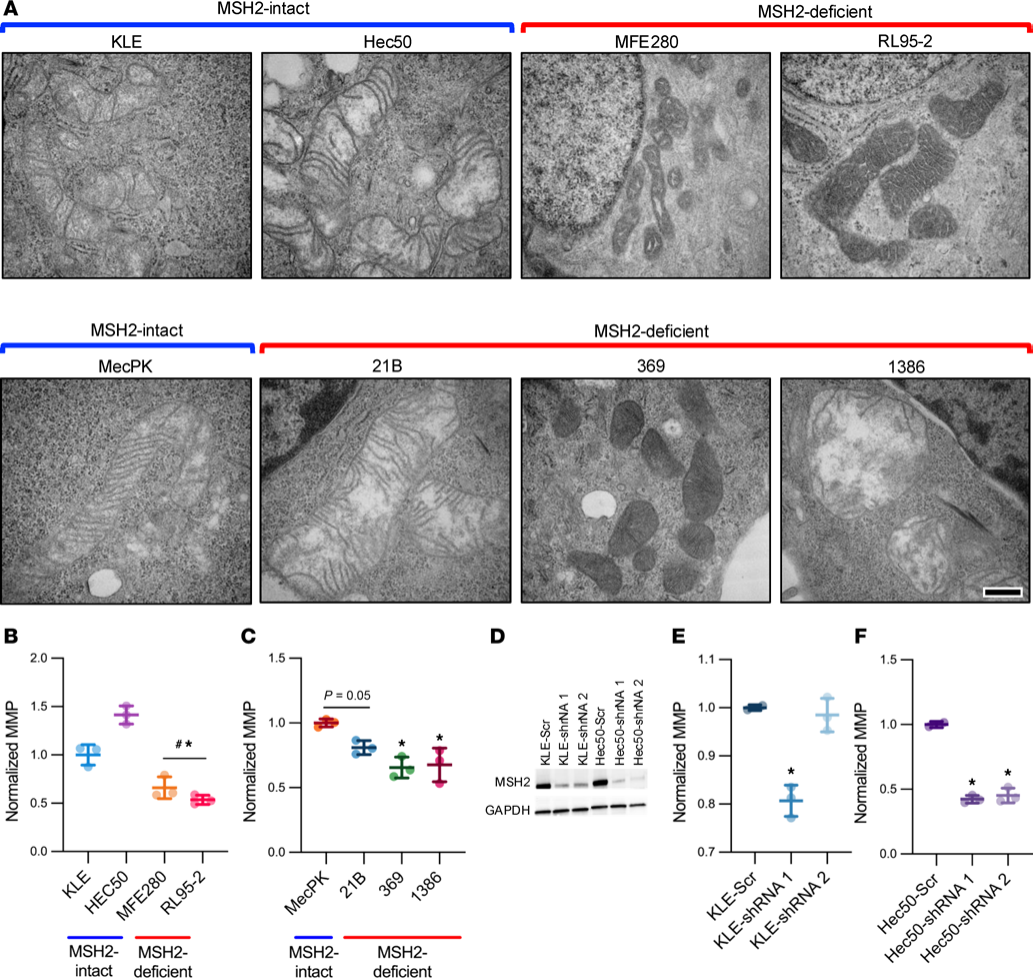
MSH2-deficient EC cells exhibit defects in mitochondrial integrity. (**A**) Transmission electron microscopy was performed on human (top row) and mouse (bottom row) MSH2-intact and MSH2-deficient EC cell lines. Scale bar: 200 nm. Mitochondrial membrane potential (MMP) was measured using flow cytometric analysis of JC-1–stained human (**B**) and mouse (**C**) EC cells (*n* = 3 technical replicates for all cell lines). Data are mean ± SD. Data points are values from individual samples. Analysis was by ANOVA with Dunnett’s multiple-comparison test. (**B**) *P < 0.05 relative to KLE; ^#^*P* < 0.05 relative to Hec50. (**C**) *P < 0.05 relative to MecPK. (**D**) Western blot of MSH2 expression (GAPDH loading control) showing lentiviral shRNA-mediated MSH2 knockdown was achieved in KLE and Hec50 cells using shRNA1 and shRNA2 sequences. MMP was measured using flow cytometric analysis of JC-1–stained KLE (**E**) and Hec50 (**F**) cells with (shRNA1, shRNA2) and without (Scr) MSH2 knockdown (*n* = 3 technical replicates for all cell lines). Data are mean ± SD. Data points are values from individual samples. Analysis was by ANOVA with Dunnett’s multiple-comparison test. (**E**) *P < 0.01 relative to KLE-Scr. (**F**) *P < 0.0001 relative to Hec50-Scr.

We next evaluated the consequences of MSH2 loss on specific changes in mitochondrial integrity and function. Transmission electron microscopy of mitochondria in MSH2-intact and -deficient EC cell lines revealed abnormalities in mitochondrial ultrastructure, including cristae malformations and mitochondrial condensation (darkened mitochondrial matrix) and swelling (enlarged mitochondrial matrix with malformed cristae) (Figure 4A). Because these ultrastructural changes are indicative of disrupted membrane polarization, we performed flow cytometric analyses of JC-1–stained cells to measure mitochondrial membrane potential (MMP) in MSH2-deficient and -intact cells. Both mouse and human MSH2-deficient EC cells exhibited reductions in MMP compared with MSH2-intact counterparts (Figure 4, B and C). Furthermore, isogenic MSH2 knockdown decreased MMP in KLE and Hec50 cells (Figure 4, D–F), although not in the KLE-shRNA2 line.

**Figure 5:**
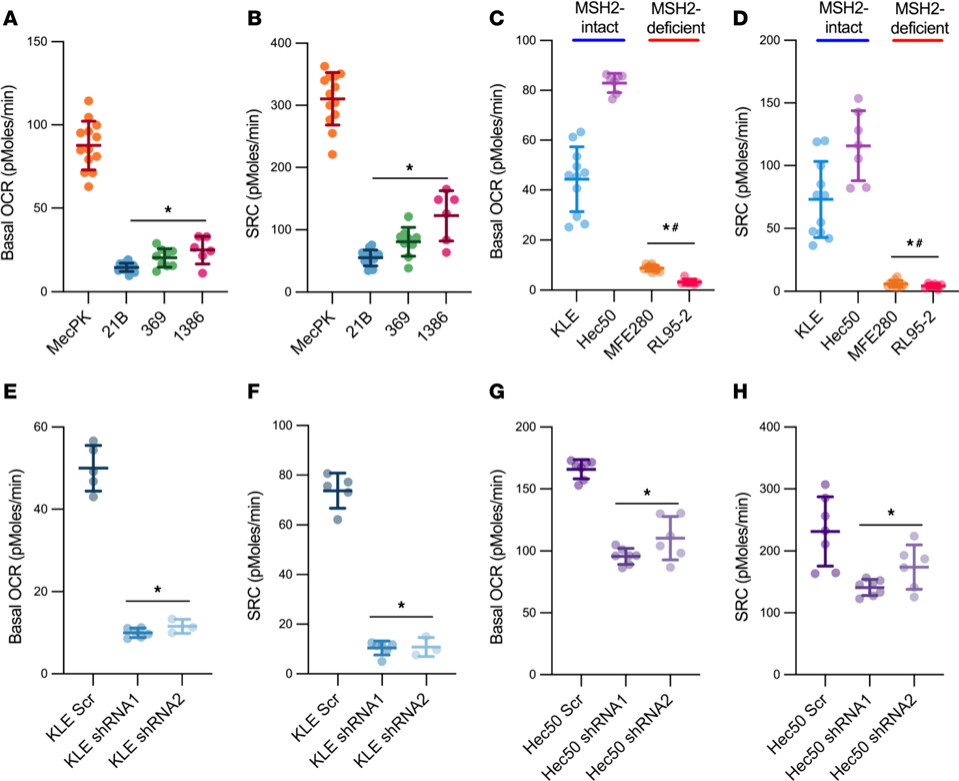
MSH2 loss conferred reduced mitochondrial function in EC cells. Mitochondrial stress tests were performed on mouse (**A** and **B**) and human (**C** and **D**) EC cells, as well as on isogenic KLE (**E** and **F**) and Hec50 (**G** and **H**) cells with and without lentiviral shRNA-mediated MSH2 knockdown. Oxygen consumption rate (OCR) was measured during mitochondrial targeting. Basal OCR (baseline OCR – oligomycin OCR) was calculated for each cell line. Spare respiratory capacity (SRC) (FCCP OCR – baseline OCR) was calculated for each cell line. *n* = 5 replicates per line. Data are mean ± SD. Data points are values from individual samples. Analysis was by ANOVA with Dunnett’s multiple-comparison test. (**A** and **B**) *P < 0.0001 relative to MecPK. (**C** and **D**) *P < 0.05 relative to KLE; ^#^*P* < 0.05 relative to Hec50. (**E**–**H**) *P < 0.05 relative to Scr controls.

Maintenance of intact MMP is essential for mitochondrial respiration, a major metabolic function of mitochondria. To measure functional consequences of defects in mitochondrial content and membrane potential as described above, we performed mitochondrial stress tests on MSH2-deficient and -intact mouse and human cells (Figure 5 and Supplemental Figure 1). MSH2-deficient mouse and human EC cells exhibited reductions in both baseline mitochondrial function (basal oxygen consumption rate [OCR]) and induced mitochondrial function (spare respiratory capacity [SRC]) compared with MSH2-intact counterparts (Figure 5, A–D). Furthermore, MSH2 knockdown decreased both baseline and induced mitochondrial function in KLE and Hec50 cells (Figure 5, E–H). Of note, all isogenic lines with MSH2 knockdown exhibited reductions in baseline and induced mitochondrial function, including the KLE-shRNA2 line that lacked differences in MMP above (see Figure 4). Based on these results, mitochondrial content, membrane potential, and respiration are disrupted in MSH2-deficient EC, causing mitochondrial dysfunction.

### Mitochondrial defects in MSH2 deficiency–related EC confers metabolic vulnerability.

To evaluate changes in the overall metabolic profile due to mitochondrial defects in MSH2-deficient EC, we performed targeted metabolomics profiling of polar metabolites using ion chromatography–mass spectrometry (IC-MS) of benign endometrial tissues from control mice (*n* = 5) and Msh2KO mice (*n* = 13) as well as EC from Msh2KO mice (*n* = 7). We compared differentially modulated metabolites in Msh2KO benign versus control benign endometrium and Msh2KO benign versus Msh2KO EC to understand potential metabolic derangements underlying MSH2-deficient carcinogenesis. We also compared Msh2KO EC versus EC from the *PR-Cre Pten^LoxP/LoxP^* mouse model (21) (abbreviated PtenKO) (*n* = 5) to understand metabolic differences that may be specific to MSH2-deficient carcinogenesis rather than a general carcinogenic phenotype. The statistically significant (FDR < 0.05) differences in metabolite abundances between Msh2KO benign and control benign are shown in Table 2, between Msh2KO benign and Msh2KO EC are shown in Table 3, and between Msh2KO EC and PtenKO EC are shown in Table 4. Many differentially modulated metabolites in Msh2KO tissues related to carbohydrate metabolism (glycolysis, fermentation, and TCA cycle), nucleotide metabolism, amino acid metabolism, and redox balance, with carbohydrate metabolism and nucleotide metabolism appearing across all 3 comparisons (22).

**Figure 6:**
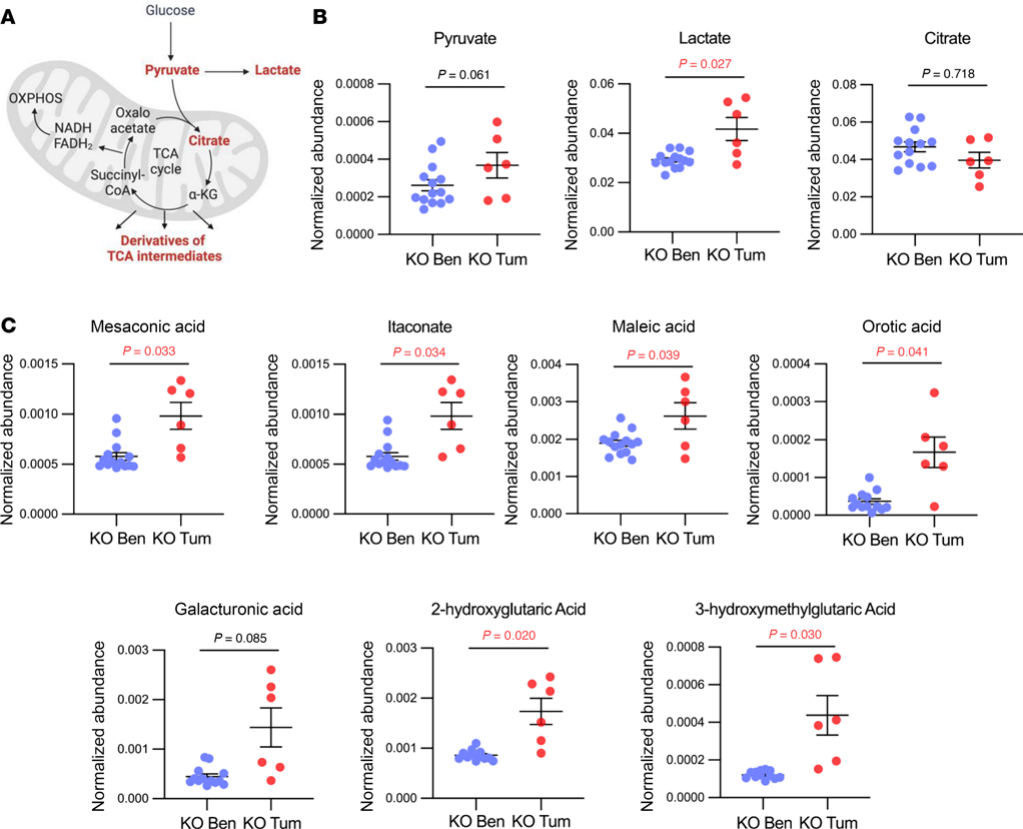
Metabolites indicative of TCA cycle dysfunction were elevated in Msh2KO EC. (**A**) Schematic overview of metabolites related to TCA cycle entry (pyruvate, lactate, citrate) and premature exit from the TCA cycle (derivatives of TCA cycle intermediates). (**B**) Quantification of relative peak metabolite abundance of those related to TCA cycle entry (pyruvate, lactate, and citrate) between Msh2KO normal endometrium (blue, *n* = 13) and Msh2KO EC (red, *n* = 7) tissues using IC-MS polar metabolomics profiling. (**C**) Quantification of relative peak metabolite abundance of derivatives of TCA cycle intermediates (mesaconic acid, itaconate, maleic acid, orotic acid, galacturonic acid, 2-hydroxyglutaric acid, 3-hydroxymethylglutaric acid) in Msh2KO normal endometrium (blue) and Msh2KO EC (red) tissues using IC-MS polar metabolomics profiling. Ben, benign; Tum, tumor. Data are mean ± SEM. Data points are values from individual mice. Analysis was by ANOVA with FDR-corrected *P* values reported in each graph.

Given that our findings suggested mitochondrial dysfunction in MSH2-deficient EC cells thus far, we evaluated further the differences in carbohydrate metabolism as it relates to entry into and exit from the TCA cycle, which takes place in the mitochondrial matrix and depends on intact mitochondrial integrity. Msh2KO tumor tissues showed significantly increased abundance of lactate compared with Msh2KO benign endometrium, suggesting that pyruvate may be converted into lactate rather than entering the TCA cycle in Msh2KO EC more so than in Msh2KO benign endometrium (Figure 6, A and B). Furthermore, Msh2KO EC exhibited significantly increased abundances of each of the measured derivatives from TCA cycle intermediates, suggesting premature departure from the TCA cycle rather than completing the cycle (Figure 6C).

**Figure 7:**
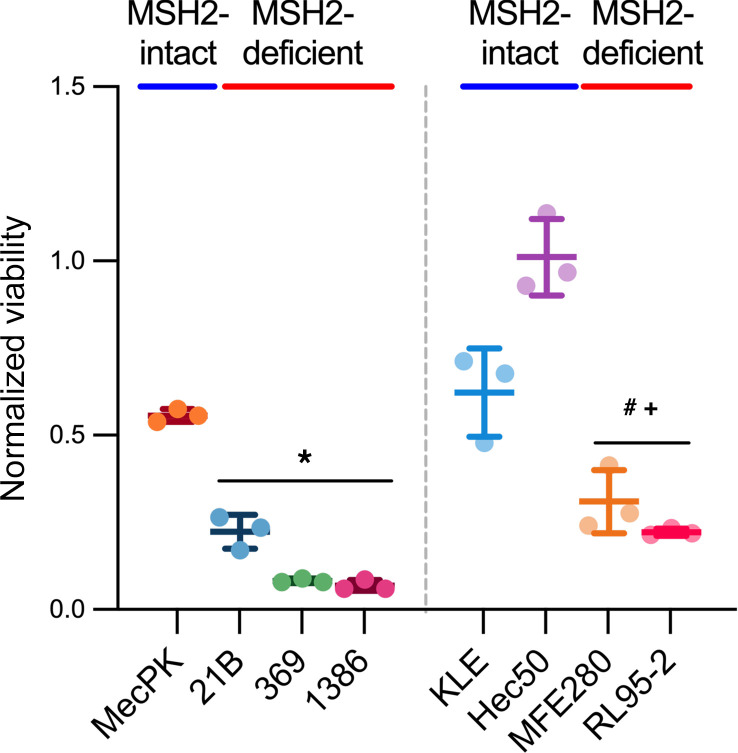
MSH2-deficient EC cells are highly vulnerable to metabolic targeting. MSH2-intact and -deficient murine and human EC cells were treated with glucose-free media supplemented with 4.5 g/L galactose for 72 hours. Viability was measured using CellTiter Glo. Viability relative to glucose-containing media (4.5 g/L) controls are shown. *n* = 3 replicates for each cell line. Data are mean ± SD. Data points are values from individual samples. Analysis was by ANOVA with Dunnett’s multiple-comparisons test. *P < 0.0001 relative to MecPK; ^#^*P* < 0.05 relative to KLE; ^+^*P* < 0.0001 relative to Hec50.

Given their mitochondrial dysfunction and evidence of metabolic reprogramming, we determined whether MSH2-deficient EC cells rely on non-mitochondrial pathways for survival. In glucose metabolism, cells can generate ATP through glycolysis followed by fermentation (e.g., producing lactate in anaerobic conditions) or by shuttling pyruvate into the mitochondria for the TCA cycle and oxidative phosphorylation. Overall, glycolysis proceeds faster than galactolysis and the surplus NADH generated from glucose preferentially reacts with pyruvate to form lactate. In galactose metabolism, the reliance on glycolysis alone for ATP production is less efficient because galactose metabolism through glycolysis yields less ATP. Thus, cells are more dependent on the TCA cycle and oxidative phosphorylation for sufficient ATP generation. The NADH formed during galactolysis is preferentially used for full oxidation within mitochondria. Therefore, treating cells with galactose-containing media in place of glucose-containing media induces cells to rely more on mitochondria for energy production (23, 24). While galactose treatment significantly reduced viability of most mouse and human EC cell lines, MSH2-deficient EC cells had significantly reduced survival after galactose treatment compared with MSH2-intact counterparts, revealing that their reliance on non-mitochondrial pathways for survival represents a metabolic vulnerability (Figure 7).

## Discussion

We report here what we believe to be the first mouse model for LS-related EC development through MSH2 loss targeted to the female reproductive tract. The Msh2KO mouse develops endometrial pathology, including AH precancerous lesions and tumors with endometrioid and non-endometrioid features similar to LS-ECs (25). This mouse model will enable mechanistic studies in LS-EC development, which will be critical in providing novel biomarkers for risk stratification and/or therapeutic targets for prevention and interception.

Pathway analyses from transcriptomic studies of Msh2KO endometrial tissues revealed mitochondrial dysfunction, and we discovered that a broad phenotype of mitochondrial content reduction may underly that finding. Furthermore, mitochondrial staining in human ECs revealed reduced mitochondrial content in MSH2-deficient tumors compared with EC tumors without MMR defects, showing that mitochondrial content depletion is not simply an artifact of carcinogenesis broadly. Retained mitochondria in MSH2-deficient EC cells have ultrastructural abnormalities in cristae and mitochondrial membranes, reduced MMP, and diminished mitochondrial respiration (oxidative phosphorylation) compared with MSH2-intact counterparts. Our studies show a strong association between MSH2-deficient EC and defects in mitochondrial content, integrity, and function. When investigating broader metabolic consequences of mitochondrial defects, differences in metabolite accumulation during EC development in Msh2KO mice suggest that impaired mitochondrial function causes metabolic reprogramming. Indeed, in vitro studies suppressing ATP production from glycolysis and fermentation to enhance reliance on oxidative phosphorylation exposed greater vulnerability of MSH2-defiicent EC cells through reduced cell viability compared with MSH2-intact EC cells.

Together, these studies indicate that diminished mitochondrial content and damage to retained mitochondria are consequences of MSH2 loss in EC and confer mitochondrial dysfunction, including reduced mitochondrial respiration and increased dependence on non-mitochondrial metabolic pathways. The mechanism for how MSH2 loss causes the mitochondrial phenotype is an area for future study. Ultimately, these findings suggest that mitochondrial dysfunction and metabolic reprogramming could be leveraged as novel biomarkers for EC development and/or targeted for cancer interception.

The observed mitochondrial phenotype as a consequence of MSH2 loss reveals that MMRd has broader effects than solely DNA hypermutability. With such knowledge and characterization of additional carcinogenic pathways in EC, new opportunities for risk stratification, surveillance, and prevention may arise. Future studies elaborating on the timing of mitochondrial defects during EC development and how they provide a selective advantage during carcinogenesis will be paramount to understanding the utility in a clinical context. Nonetheless, this research represents an important discovery of the association between LS-EC and mitochondrial defects.

To evaluate timing of mitochondrial defects during EC development, measuring mitochondrial content in tissue specimens from endometrial samples throughout the EC development spectrum would be necessary. Furthermore, longitudinal sampling, such as from patients with LS who have surveillance biopsies, would allow for evaluation of mitochondrial content over time with potential progression to precancerous lesions or EC. Lastly, comparisons between benign and/or precancerous tissues from patients with and without LS would aid in determining specificity and timing of mitochondrial aberrations during LS-EC development. Importantly, such tissue-based studies would capture potential heterogeneity of mitochondrial dysfunction related to MSH2 loss, which is not reflected in our in vitro and in vivo modeling systems, as well as possible mitochondrial differences between endometrioid and non-endometrioid carcinogenic pathways. Additionally, future quantitative and spatial mapping of mitochondrial networks in tumors (26) will provide exciting opportunities to provide further insights into the metabolic and adaptive phenotype in MSH2-deficient tumorigenesis.

Our study evaluated MSH2-deficient EC to determine additional mechanisms for LS-EC development. However, whether the mitochondrial aberrations are MSH2 specific or relevant to LS more broadly remains to be studied. Additionally, evaluation of MMRd via MLH1 deficiency (most commonly via hypermethylation) is an area for future study.

How MSH2 deficiency or MMRd leads to mitochondrial content reduction is an important area for further studies. MSH2 loss and/or MMRd may lead to increased mutations in nuclear-encoded mitochondrial genes. However, we did not observe convergent mutations in nuclear-encoded mitochondrial genes in MSH2-deficient ECs in our study. Another potential mechanism could be hypermethylation of nuclear-encoded mitochondrial genes. Although similar studies are lacking in EC, LS-related colorectal cancers exhibit broad epigenetic changes (27), lending rationale to this hypothesis. Lastly, questions arise regarding whether MSH2 and other MMR proteins participate in mitochondrial DNA repair, as with nuclear DNA repair. Limited studies have evaluated mammalian DNA MMR protein function in the mitochondria, but colocalization studies have found that while MLH1 appears to colocalize with mitochondria and may participate in mitochondrial DNA repair, MSH2 does not (28–30). Sequencing mitochondrial DNA would aid in understanding whether deficient mitochondrial DNA repair may underly our findings. Finally, rather than a direct relationship between MMR proteins and mitochondria, the mitochondrial phenotype could be an indirect consequence of MMRd. Understanding the underlying mechanism whereby MSH2 loss leads to mitochondrial dysfunction will be imperative in determining opportunities for therapeutic targeting in EC prevention and interception.

The mitochondrial phenotype we identified in MSH2-deficient EC may represent a broader signal of stress rather than simply a direct consequence of MSH2 loss. Mitochondrial content reduction occurs in the setting of ongoing cellular stressors, including due to prolonged exposure to elevated glucocorticoids, insulin, and oxidative stress (as reviewed in ref. 31), which are risk factors in EC development. Perhaps chronic activation of cellular stress pathways in the setting of MSH2 deficiency would contribute to mitochondrial content reduction under similar mechanisms. Such mitochondrial reduction contributes to reduced “resilience” to additional stressors and could contribute to the multiplicative carcinogenic risk of other EC risk factors (such as conditions that increase exposure to unopposed estrogen, obesity, and/or insulin resistance) in addition to LS (32). Likewise, estrogen is known to promote mitochondrial biogenesis (as reviewed in ref. 15), suggesting future studies to assess the effects of chronic hyperestrogenism on mitochondrial biology in the endometrium as well as its interplay with MSH2 deficiency. Viewing mitochondrial reduction as a phenotype of accumulated “risk” calls for investigation of mitochondrial content changes in other prolonged risk conditions for EC, such as obesity. Excitingly, this would establish a new opportunity for biomarker-based risk stratification in EC based on mitochondrial factors.

In conclusion, this research shows mitochondrial aberrations due to MSH2 loss in LS-related EC development. These studies reveal that mitochondrial dysfunction is a noncanonical consequence of MMRd in LS-related endometrial carcinogenesis. Biomarkers for mitochondrial defects may be useful in screening or risk stratification purposes for EC. Furthermore, metabolic vulnerability represents a key opportunity that may be leveraged for preventive or therapeutic purposes. Ultimately, these findings add to our understanding of endometrial carcinogenesis and support rationale for future studies on mitochondrial biology in EC.

## Methods

### Sex as a biological variable.

Our study exclusively examined female mice because the disease modeled is only relevant in females. Likewise, human tissue studies were from only female patients.

### Generation of PR-Cre Msh2^LoxP/LoxP^ mice.

PR Cre knockin (*PR-Cre*) mice were developed previously (19) and were gifted by John Lydon (Baylor College of Medicine, Houston, Texas, USA). *Msh2^LoxP/LoxP^* mice were developed previously (20) and shared by Raju Kucherlapati (Harvard Medical School, Boston, Massachusetts, USA).

*Msh2^LoxP/LoxP^* mice were crossed with *PR-Cre* mice to generate *PR-Cre Msh2^LoxP/+^* mice, and then intercrossed to create *PR-Cre Msh2^LoxP/LoxP^* mice (termed Msh2KO). Female breeders were *Msh2^LoxP/LoxP^* mice lacking *PR-Cre*. Control mice have the *Msh2^LoxP/LoxP^* gene but lack the *PR-Cre* recombinase. Offspring from these crosses were genotyped by PCR using previously established methods (19, 20) and backcrossed to wild-type C57BL/6J mice (Jackson Laboratory). SNP typing was utilized for confirmation of the C57BL/6J background using the Genetically Engineered Mouse Facility (MD Anderson Cancer Center).

### Histologic profiling of mouse tissues.

Mice were humanely sacrificed and uterine tissues were harvested, fixed in 10% formalin, and embedded in paraffin. Formalin-fixed, paraffin-embedded (FFPE) uterine tissues stained with hematoxylin and eosin (H&E) were examined by a veterinary pathologist for histologic determination. Msh2KO tumors were reviewed by a clinical gynecologic pathologist to categorize histologic subtypes similar to human ECs.

### Human sample studies.

FFPE tumors resected from patients with EC were obtained from the University of Texas MD Anderson Cancer Center Gynecologic Oncology Tissue Bank. Relevant clinical information for human tissues utilized can be found in Supplemental Table 4.

### IHC.

For IHC staining, slides were stained using the Leica Bond Rx Autostainer system. Tissue sections were labeled as single IHC stains for antigens using an anti-MSH2 antibody (2017S, Cell Signaling Technology) or anti-TOM20 antibody (42406, Cell Signaling Technology). Slides were dewaxed in Bond Dewax solution (AR9222, Leica Biosystems) and hydrated in Bond Wash solution (AR9590, Leica Biosystems). Heat-induced antigen retrieval was performed at 100ºC in Bond-Epitope Retrieval solution 1 pH 6.0 (AR9961, Leica Biosystems). After pretreatment, slides were incubated for 1 hour with MSH2 at 1:300 or TOM20 at 1:200 followed by ready-to-use Novolink Polymer (RE7260-K, Leica Biosystems) secondary antibody. Antibody detection with 3,3′-diaminobenzidine (DAB) and hematoxylin counterstain were performed using the Bond Intense R detection system (DS9263, Leica Biosystems). Stained slides were dehydrated and coverslipped with Cytoseal 60 (23-244256, Thermo Fisher Scientific). A positive control and negative control (no primary control) slide was included for this assay. IHC stained slides were digitally imaged in Aperio AT2 (Leica) using an ×20 objective. For TOM20 quantitation, regions of interest were annotated in Aperio ImageScope software then analyzed using the Cytoplasmic v2 algorithm in the software to determine percentage TOM20^hi^ and percentage TOM20^lo^ cells, with a signal intensity threshold for high expression at 162 (defined as 3+ in the Cytoplasmic v2 algorithm).

### Transcriptomic profiling of mouse tissues.

Harvested uterine tissues from Msh2KO and control mice were embedded in optimal cutting temperature compound (OCT) and rapidly frozen on dry ice. Tissue sections were fixed in 70% ethanol and then microdissected using laser-capture microdissection technology, which enables isolation of regions of interest. Due to the microscopic tumor size for some lesions, RNA yield was relatively limited. After RNA isolation, samples were profiled using the Mouse Clariom D assay (Affymetrix), a microarray-based broad transcriptome-level expression profiling system that can be performed using low-input specimens.

### Cell line development and culture information from Msh2KO mice.

Two-dimensional cell lines have been established from tumor tissue from Msh2KO mice. These 3 cell lines (21B, 369, and 1386) were developed from histologically and molecularly distinct tumors and enable mechanistic study of pathways underlying Msh2KO cancer signaling. We compared Msh2KO cell lines in vitro to a MecPK cell line (gifted by Jae-Wook Jeong, University of Missouri, Columbia, Missouri, USA), derived from a high-grade endometrial tumor that lacked PTEN expression from the MecPK (*Pten-*null, *Kras-*mutant) mouse model (33). The 21B cell line was derived from an Msh2KO low-grade uterine tumor with endometrioid-like histology that lacked PTEN expression. The 369 cell line was derived from an Msh2KO high-grade uterine tumor with mixed endometrioid- and serous-like histology and intact PTEN expression. The 1386 cell line was derived from an Msh2KO low-grade uterine tumor with endometrioid-like histology and with intact PTEN expression. Similar to MecPK, Msh2KO cell lines do not express estrogen receptor (Supplemental Figure 2).

Primary mouse cell lines (MecPK, 21B, 369, and 1386) were maintained in DMEM supplemented with 10% FBS (HyClone), 10 mL/L penicillin/streptomycin solution (Gibco), and 100 mg/mL Primocin (Invivogen). Cell lines were authenticated and tested for mycoplasma using the Characterized Cell Line Core Facility at MD Anderson Cancer Center.

### Human cell culture.

Human cell lines were purchased from American Type Culture Collection (ATCC) or European Collection of Authenticated Cell Cultures (ECACC). KLE and RL95-2 cells were maintained in DMEM/F12 supplemented with 10% FBS, 10 mL/L penicillin/streptomycin solution, and 100 mg/mL Normocin (Invivogen). Hec50 and MFE-280 cells were maintained in MEM supplemented with 10% FBS, 10 mL/L penicillin/streptomycin solution, and 100 mg/mL Normocin.

### IHC/immunofluorescent staining of cells in vitro.

Cells were seeded onto 0.17-mm-thick (no. 1.5) glass coverslips (Warner Instruments) precoated overnight with 60 μg/mL collagen I (Gibco), then fixed using freshly prepared 4% paraformaldehyde (Electron Microscopy Sciences) in PBS for 15 minutes at room temperature, and then washed in PBS. Background Sniper (Biocare Medical) was used to block nonspecific staining, and then anti-TOM20 rabbit mAb (D8T4N, Cell Signaling Technology) was diluted 1:100 in Background Sniper and applied to coverslips overnight at 4°C. Following PBS wash, coverslips were incubated in goat anti-rabbit IgG (H+L) highly cross-adsorbed Alexa Fluor Plus 488–conjugated secondary antibody (A32731, Invitrogen) at a dilution of 1:1000 in Background Sniper for 1 hour at room temperature. Coverslips were washed and then counterstained with HCS CellMask Deep Red (Thermo Fisher Scientific; 1:5000 in PBS) and Hoechst 33342 solution (Invitrogen; 1 μg/mL in PBS) for 30 minutes at room temperature. Coverslips were washed and then mounted in Prolong Gold Anti-Fade Mounting Media (Invitrogen) on glass slides and dried overnight.

Imaging of stained cells was conducted with a ×63/1.46 NA oil objective on the Zeiss Laser-point Scanning Microscope (LSM) 880 with Airyscan module for ultra-high-resolution imaging in the Advanced Microscopy Core at MD Anderson Cancer Center. Raw.czi files were processed using the Zen software into deconvoluted Airyscan images and analyzed using CellProfiler (Broad Institute; https://cellprofiler.org/). TOM20 intensity was quantified relative to cell area.

### Transmission electron microscopy.

Cells seeded on tissue culture–treated plates were fixed with 3% glutaraldehyde and 2% paraformaldehyde in 0.1 M cacodylate buffer (pH 7.3), and then washed in 0.1 M sodium cacodylate buffer. Processing and imaging took place at the High Resolution Electron Microscopy Core Facility at MD Anderson Cancer Center. Samples were treated with 0.1% Millipore-filtered cacodylate-buffered tannic acid, postfixed with 1% buffered osmium tetroxide, and stained en bloc with 0.1% Millipore-filtered uranyl acetate. The samples underwent serial dehydration in increasing concentrations of ethanol then infiltrated and embedded in LX-112 medium. The samples underwent polymerization in a 60°C oven for 72 hours, and ultrathin sections were cut using Leica Ultracut microtome. Sections were mounted on formvar-coated single-slot copper grids and stained with uranyl acetate and lead citrate in a Leica EM Stainer. The stained samples were examined in a JEM 1010 transmission electron microscope (JEOL USA, Inc.) using an accelerating voltage of 80 kV. Digital images were obtained using an Advanced Microscopy Techniques imaging system (Advanced Microscopy Techniques Corp).

### Lentivirus infection for MSH2 knockdown.

KLE and Hec50 cells were transduced with lentivirus containing a scrambled shRNA sequence or 1 of 2 shRNA sequences complementary to the *MSH2* mRNA. Lentivirus containing *U6* promoter–based shRNA vectors were purchased from VectorBuilder.

On day 1, cells were trypsinized and resuspended in growth media at a density of 200,000 cells/mL. Polybrene (VectorBuilder) was added to the cell suspension at a volume of 1:1000 and virus was added at a multiplicity of infection (MOI) of 10 transducing units per cell. Each 1 mL of suspension containing cells, polybrene, and virus was seeded in 1 well of a 6-well tissue culture dish. On day 2, virus-containing media were discarded and replaced with fresh growth media. On day 3, growth media were replaced with media containing puromycin (10 μg/mL) for antibiotic selection. Selection was confirmed by visualizing fluorescence of cells infected with an EGFP-expressing control virus.

### MMP flow cytometric assay.

Live cells were trypsinized, washed, and then stained with JC-1 (Invitrogen), a cationic carbocyanide dye that accumulates in mitochondria. JC-1 exists as a monomer at low concentrations and yields green fluorescence. At higher concentrations, JC-1 aggregates form that exhibit red fluorescence. JC-1 accumulation in mitochondria is MMP dependent, so the JC-1 flow assay serves as a sensitive marker for MMP. Cells were stained with JC-1 at a concentration of 10 μg/mL in Live Cell Imaging Solution (Invitrogen) at 37°C for 10 minutes with agitation. Following washing with PBS, cells were analyzed using flow cytometry at the Flow Cytometry and Cellular Imaging Core Facility at MD Anderson Cancer Center. Fl1 and Fl2 fluorescence was measured on the Beckman Coulter Gallios. The Fl1 to Fl2 ratio represents the MMP.

### Mitochondrial stress tests.

Cells were seeded on Seahorse XFe96 cell culture microplates (Agilent). On the day of the assay, cells were equilibrated for 1 hour in XF Base Assay Medium supplemented with 10 mM glucose, 1 mM sodium pyruvate, and 2 mM L-glutamine in a CO_2_-free incubator at 37°C. Oligomycin (1.5 μM), carbonyl cyanide-4 (trifluoromethoxy) phenylhydrazone (FCCP) (1 μM), and rotenone A/antimycin (0.5 μM) were loaded into injection ports on the prehydrated Seahorse XFe96 sensor cartridge and loaded into the Seahorse XFe96 bioanalyzer for calibration. The microplate was then loaded into the Seahorse XFe96 bioanalyzer for analysis using the XF Cell Mito Stress Test assay template file in Wave software. Parameters of interest are automatically calculated in the Seahorse XF Mito Stress Test Report Generator from Wave data and exported to Microsoft Excel. All parameters of interest were normalized for cell number across experiments.

### Baseline metabolomics profiling by IC-MS.

Flash-frozen uterine tissues from Msh2KO and control mice (aged 14–16 months) or tumors from the *PR-Cre Pten^LoxP/LoxP^* mouse model (PtenKO) for EC (21) that developed tumors by 8 weeks of age (gifted by Jae-Wook Jeong, University of Missouri) were processed by homogenizing with a Precellys Tissue Homogenizer and metabolites were extracted in ice-cold 0.1% ammonium hydroxide in 80:20 (v/v) methanol/water. Extracts were centrifuged at 17,000*g* for 5 minutes at 4°C and supernatants were transferred to clean tubes and dried by evaporation under nitrogen. Dried extracts were reconstituted in deionized water. Samples of 5 μL were injected for analysis by IC-MS. IC mobile phase A (MPA; weak) was water, and mobile phase B (MPB; strong) was water containing 100 mM KOH. A Thermo Fisher Scientific Dionex ICS-6000+ system included a Thermo IonPac AS11 column (4 μm particle size, 250 × 2 mm) with the column compartment maintained at 35°C. The autosampler tray was chilled to 4°C. The mobile phase flow rate was 350 μL/min and a gradient from 1 mM to 100 mM KOH was used. The total run time was 55 minutes. To assist the desolvation for better sensitivity, methanol was delivered by an external pump and combined with the eluent via a low-dead-volume mixing tee. Data were acquired using a Thermo Orbitrap IQ-X Tribrid mass spectrometer under ESI negative ionization mode at a resolution of 240,000. Raw data files were imported to Thermo Trace Finder 5.1 software for final analysis.

The peak area (raw relative abundance) of each metabolite was normalized by dividing by the total peak area of the respective sample to generate normalized metabolite relative abundance values.

### Galactose experiments in vitro.

Cells were treated for 72 hours with glucose-free medium supplemented with 4.5 g/L galactose (Sigma-Aldrich) or 4.5 g/L glucose (Sigma-Aldrich) as the vehicle control. Cell viability was measured using CellTiter Glo 2.0 (Promega).

### Statistics and data analyses.

All statistical methods were performed in GraphPad Prism version 7 unless otherwise indicated. Comparisons of continuous variables between 2 groups were performed using 2-tailed Student’s *t* tests. Comparisons of continuous variables between greater than 2 groups was performed using 1-way ANOVA with post hoc Tukey or Dunnett’s test as indicated. Nonparametric alternatives for *t* tests (Mann-Whitney *U* test) or ANOVA (Kruskal-Wallis test) were used as indicated where data were not expected to follow normal distribution. The Benjamini-Hochberg method was employed to correct for multiple hypothesis testing as indicated.

### Study approval.

Mouse studies were approved by the IACUC at the University of Texas MD Anderson Cancer Center. Human tissues were obtained from the University of Texas MD Anderson Cancer Center Gynecologic Oncology Tissue Bank after obtaining written informed consent under protocols approved by the Institutional Review Board.

### Data availability.

Values for all data points in graphs are reported in the Supporting Data Values file. Transcriptomic data from mouse studies have been deposited in the NCBI GEO under accession GSE288518 (https://www.ncbi.nlm.nih.gov/geo/query/acc.cgi?acc=GSE288518).

## Author contributions

MBB developed methodology, conducted experiments, verified replication and reproducibility of results/experiments, generated figures, and analyzed and curated data. BM developed methodology, provided software, conducted experiments, verified replication and reproducibility of results/experiments, and analyzed and curated data. QZ, KD, and JC developed methodology, conducted experiments, and provided resources. DM, LP, and NG developed methodology and conducted experiments. WKC developed methodology, conducted experiments, analyzed data, and provided resources. CJ developed methodology and provided software and resources. LT developed methodology, provided software, conducted experiments, provided resources, and analyzed data. MAZ conducted experiments and provided resources. PLL provided resources and supervision. RKY and RBB conducted experiments, verified replication and reproducibility of results/experiments, and provided resources. EW developed methodology, conducted experiments, verified replication and reproducibility of results/experiments, and provided resources. DMS developed methodology, conducted experiments, and analyzed data. THK and JWJ developed methodology and provided resources. RS developed methodology, conducted experiments, and curated data. KL conceptualized and supervised the study and acquired funding. HEK developed methodology and provided supervision. MSY conceptualized the study, developed methodology, provided resources, project administration, and supervision, wrote the original draft of the manuscript, and acquired funding. All authors reviewed and edited the manuscript.

## Supplementary Material

Supplemental data

Unedited blot and gel images

Supplemental table 1

Supplemental table 2

Supplemental table 3

Supporting data values

## Figures and Tables

**Table 1:**
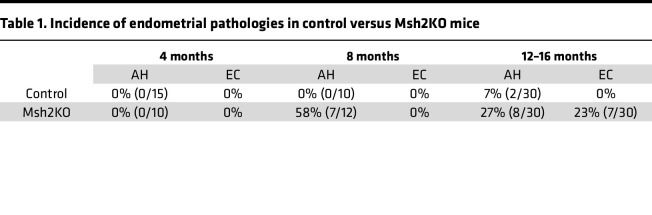
Incidence of endometrial pathologies in control versus Msh2KO mice

**Table 2:**
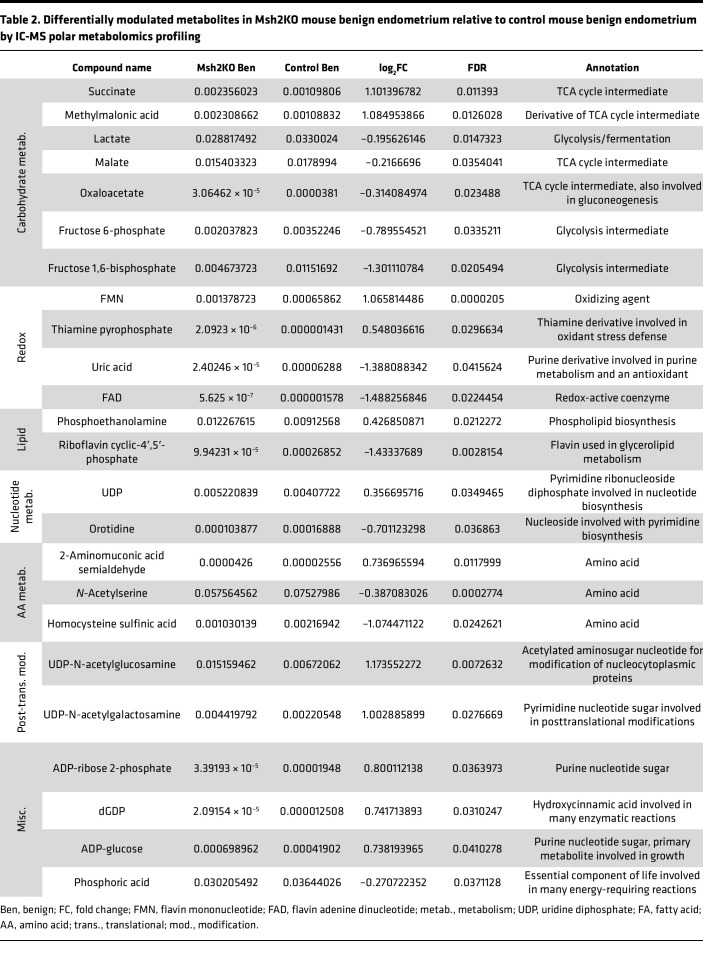
Differentially modulated metabolites in Msh2KO mouse benign endometrium relative to control mouse benign endometrium by IC-MS polar metabolomics profiling

**Table 3:**
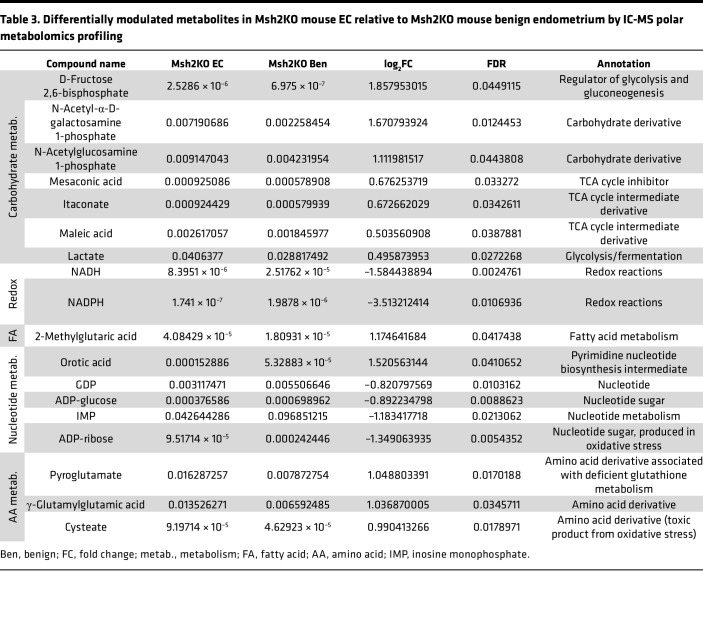
Differentially modulated metabolites in Msh2KO mouse EC relative to Msh2KO mouse benign endometrium by IC-MS polar metabolomics profiling

**Table 4:**
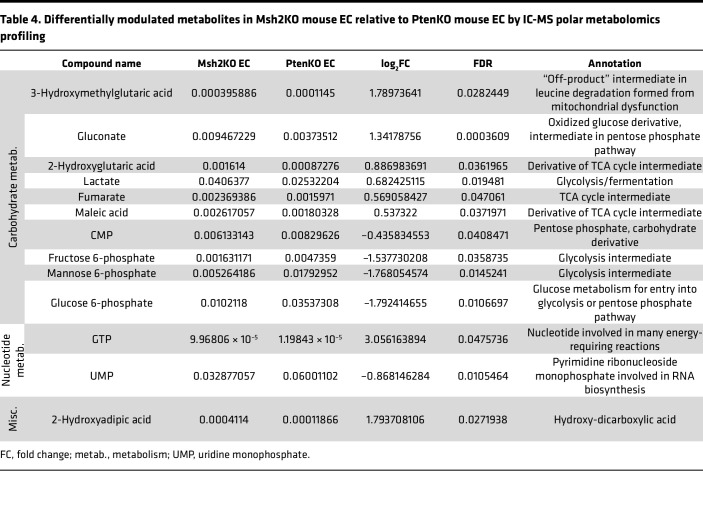
Differentially modulated metabolites in Msh2KO mouse EC relative to PtenKO mouse EC by IC-MS polar metabolomics profiling

## Version 1 02/18/2025

In-Press Preview

## Version 2 03/24/2025

Electronic publication
