# Supplementary figures and images for: Mitochondrial defects and metabolic vulnerabilities in Lynch syndrome–associated MSH2-deficient endometrial cancer
Source: JCI Insight. 2025 Feb 18;10(6):e185946. doi: 10.1172/jci.insight.185946 (PMC11949016; doi:10.1172/jci.insight.185946)

Supplemental Figure 1

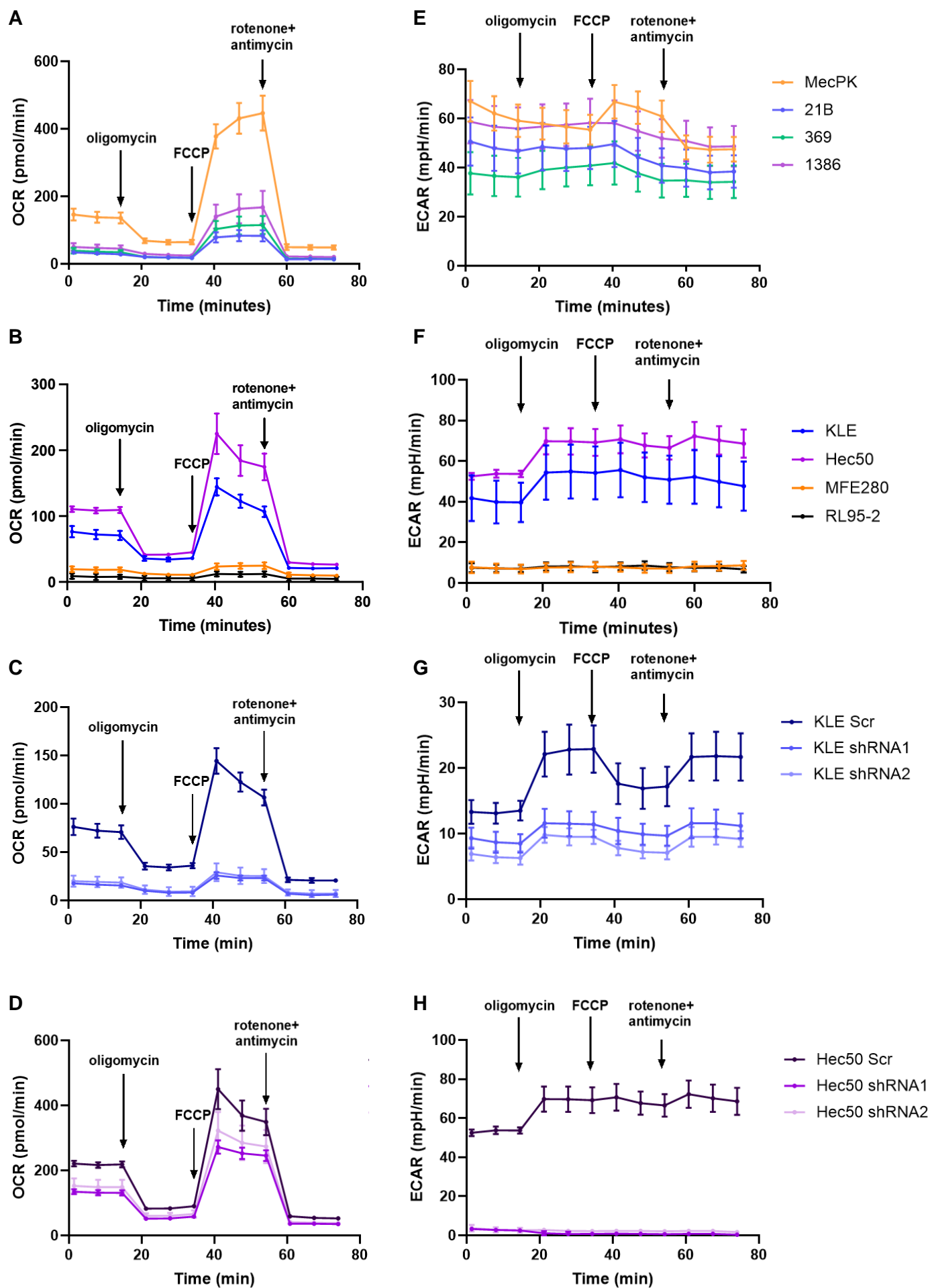

Supplemental Figure 2

A

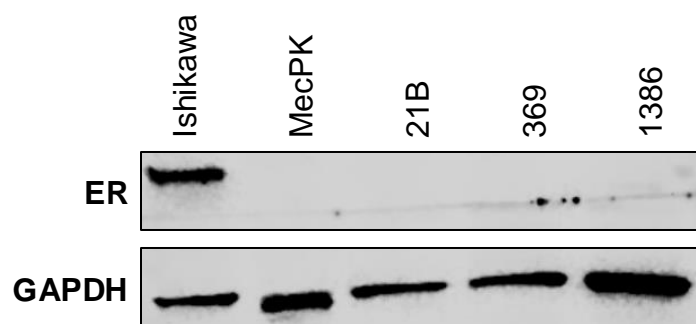

B

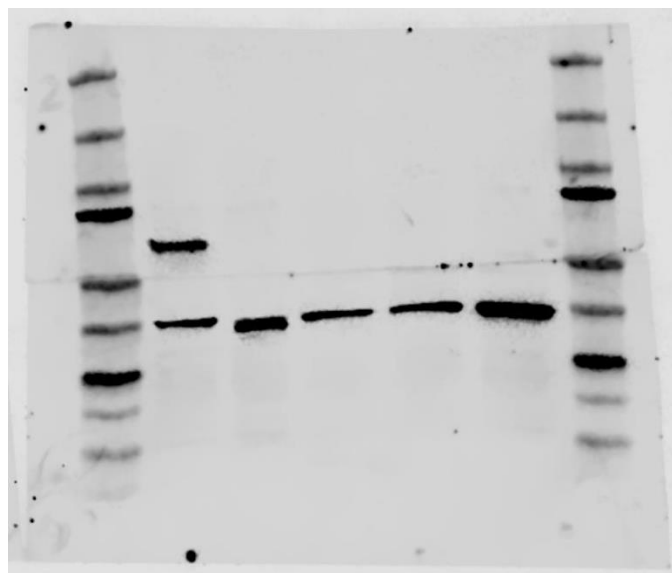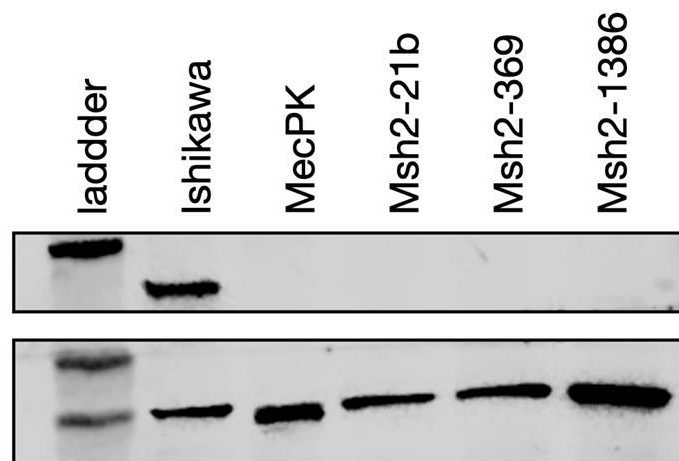

Supplement: Supplemental data [file jciinsight-10-185946-s268.pdf]

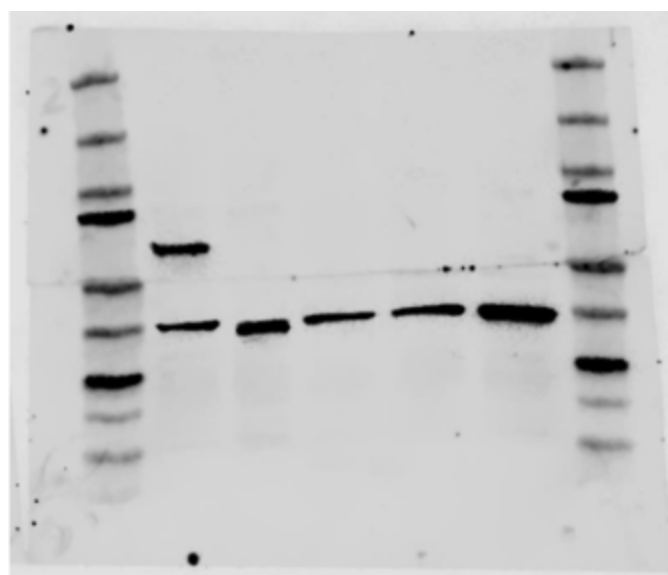

Supplement: Unedited blot and gel images [file jciinsight-10-185946-s269.pdf]
